# RFE-UNet: Remote Feature Exploration with Local Learning for Medical Image Segmentation

**DOI:** 10.3390/s23136228

**Published:** 2023-07-07

**Authors:** Xiuxian Zhong, Lianghui Xu, Chaoqun Li, Lijing An, Liejun Wang

**Affiliations:** College of Information Science and Engineering, Xinjiang University, Urumqi 830049, China; zhxx@xju.edu.cn (X.Z.); xlh@stu.xju.edu.cn (L.X.); lcq@stu.xju.edu.cn (C.L.); alj@stu.xju.edu.cn (L.A.)

**Keywords:** U-Net, transformer, remote feature exploration

## Abstract

Although convolutional neural networks (CNNs) have produced great achievements in various fields, many scholars are still exploring better network models, since CNNs have an inherent limitation—that is, the remote modeling ability of convolutional kernels is limited. On the contrary, the transformer has been applied by many scholars to the field of vision, and although it has a strong global modeling capability, its close-range modeling capability is mediocre. While the foreground information to be segmented in medical images is usually clustered in a small interval in the image, the distance between different categories of foreground information is uncertain. Therefore, in order to obtain a perfect medical segmentation prediction graph, the network should not only have a strong learning ability for local details, but also have a certain distance modeling ability. To solve these problems, a remote feature exploration (RFE) module is proposed in this paper. The most important feature of this module is that remote elements can be used to assist in the generation of local features. In addition, in order to better verify the feasibility of the innovation in this paper, a new multi-organ segmentation dataset (MOD) was manually created. While both the MOD and Synapse datasets label eight categories of organs, there are some images in the Synapse dataset that label only a few categories of organs. The proposed method achieved 79.77% and 75.12% DSC on the Synapse and MOD datasets, respectively. Meanwhile, the *HD*95 (mm) scores were 21.75 on Synapse and 7.43 on the MOD dataset.

## 1. Introduction

In recent years, with the rapid development of computer vision technology, medical image analysis has been widely used in disease diagnosis and treatment planning. Furthermore, medical image segmentation as a part of the medical image analysis process has received more attention.

Medical image segmentation is often the first step in medical image analysis, and it plays an essential role in computer-aided quantitative analysis. Since CNNs have been applied to medical image segmentation tasks, U-shaped structures based on encoders, decoders, and their variants [1,2,3,4] have shown excellent performance in various medical image segmentation tasks [5,6,7]. For example, U-Net [1] has achieved good results in the heart segmentation dataset obtained by Magnetic Resonance (MR) technology [8], the multi-organ segmentation dataset obtained by Computed Tomography (CT) technology [9,10,11], and the polyp segmentation dataset obtained by colonoscopy video [12]. To date, many of the best medical segmentation network architectures have been based on U-Net.

Although the medical segmentation architecture based on CNNs has achieved good segmentation results in different types of medical datasets, these networks have a common defect; that is, it is difficult for convolutional kernels to conduct remote modeling of features when extracting semantic information for images. If the distance between different foreground elements in an image is considerable, relying solely on convolution kernels [13] for feature extraction can create a challenge for the network to understand the interrelation between those foreground elements. Due to the inherent limitations of convolution kernels, several researchers have attempted to mitigate this issue by employing dilated convolution kernels [14,15,16], which expand the receptive field of elements. However, the dilated convolution kernel is conducted on the basis of discarding some elements, so this method needs improvement.

In addition, some scholars have tried to apply transformers to CNNs to improve the insufficient long-distance modeling capability of convolutional kernels [17,18,19]. Transformers have initially shown excellent performance in natural language processing (NLP) tasks [20,21]. This is because the self-attention module within a transformer can compute the correlation coefficient between each element within a feature graph and all other elements. Through a relevance coefficient, the network can assign an appropriate weight to each element to enhance the importance of foreground information. Therefore, transformers have strong remote modeling capabilities [22,23]. For example, CA-Net [24] is based on a transformer and V-Net, which can learn contextual information from each slice to achieve automatic segmentation of the left atrium. Therefore, many scholars are trying to mitigate the insufficient remote modeling of convolutional kernels by using transformers in encoders [22,25,26]. Transformers can also be transferred to downstream applications [27]. In this context, some medical image segmentation works [26,28] have also achieved satisfactory results, and pure transformer models [29] have emerged.

To sum up, both convolution kernels and transformers have their own advantages and disadvantages. Of these, the convolutional kernel excels at learning local details, but has certain limitations in its ability to model remote features. Meanwhile, the transformer has great global modeling capabilities, but at the expense of local details. In addition, the transformer often relies on large amounts of data to demonstrate its powerful global context modeling capability. Due to various restrictions in real life, medical imaging data are often difficult to obtain in such large quantities, which limits the performance of transformer in medical image segmentation tasks. Furthermore, when it comes to medical images, foreground information is typically present in the form of local patches, and various types of foreground information tend to be spatially distant from one another. Thus, to enhance the network’s segmentation capability for foreground information, it is crucial for the network to consider correlations between the local details of the feature map and the distant elements. To address these challenges, this paper proposes a novel module called remote feature exploration (RFE). This module can use remote elements to assist in the generation of local features, which, to a certain extent, provides the network with both local detail information extraction and a remote modeling capability.

In summary, this paper delivers two main contributions:

(1) In this paper, a new multi-organ segmentation dataset is created, and the advantages and disadvantages of both the convolution operation and the transformer are verified.
(2) In this paper, we propose that the remote feature exploration layer can be used to assist the network in learning local elements using remote elements. This capability allows the network to not only capture local details but also model the relationships between distant elements.

## 2. Related Work

Early medical image segmentation methods included traditional algorithms that relied on machine learning and contour-based techniques. With the development of depth convolution neural networks (ConvNets), U-Net [1] was proposed for medical image segmentation. U-Net [1] is a symmetric codec structure that dominates the field of medical image segmentation with excellent performance. However, the skip connections in U-Net [1] directly combine the shallow features of the encoder with the deep features of the decoder, resulting in a semantic gap. Therefore, more robust variants based on U-Net [1] networks were created. For example, UNet++ [12], which has nested and dense skip connections, alleviates the generation gap between U-Net layers to a certain extent, and produces significant performance gains compared with U-Net [1]. However, UNet++ [12] cannot capture the semantic features at full scale. Attention U-Net [30] adds an integrated attention gate on the basis of U-Net [1], which can eliminate the response of redundant ambiguity in skip connections. In addition, there are many models based on neural networks, such as R50 U-Net [31], R50 Att-UNet [31], DARR [32], and UNet3+ [33]. In medical image segmentation, a U-shaped network is also used for 3D image segmentation, such as V-Net [34], which uses a CNN to train end-to-end and directly process 3D NMR. Meanwhile, 3D U-Net [35] uses elastic deformation to dynamically expand data in real-time so that the network can learn more images during each training iteration.

The transformer originates from machine translation and text embedding. In addition, the transformer has achieved competitive performance in many computer vision tasks, such as image recognition, target detection, semantic segmentation, real-world segmentation, image classification, and medical image segmentation. In the field of computer vision, a typical network based on the transformer model is the Vision Transformer (ViT) [22]. The ViT outperforms the CNN on recognition tasks, although this is achieved at the cost of a large dataset. Chen et al. designed TransUNet [26] based on a ViT, which explored the potential of a transformer in medical image segmentation using a transformer as the last encoder layer in the encoder part. A series of network architectures combining a transformer with a CNN in the encoder part, such as MedT [36] and pmTrans [37], were subsequently proposed to achieve better feature modeling. However, directly using encoders based on the transformer combined with the CNN to construct global modeling creates significant computational complexity, which increases the difficulty of designing the transformer on high-resolution feature maps. To improve the computational efficiency on high-resolution feature maps, SwinUNet [29] performs correlation modeling within a series of moving windows. Although the above architectures reduce the computational complexity of the models, there is still a significant local feature loss.

Tang et al. [38] designed a hybrid self-supervised agent task, including rotation prediction, instance antagonism, and inpainting, and demonstrated the effectiveness of the method through the effect of fine-tuning. Similarly, UNETR [39] uses a pure transformer as an encoder to learn the sequential representation of input quantities and effectively capture global multi-scale information, while also following the U-shaped structure of the encoder and decoder. PHTrans [40] mixes a transformer and a CNN in parallel as major components to generate hierarchical representations and adaptive aggregation from global and local features. However, unlike the above methods, our model can not only complete global modeling, but also uses remote elements to help generate local information, achieving an accurate segmentation effect.

## 3. Methods

**Figure 1:**
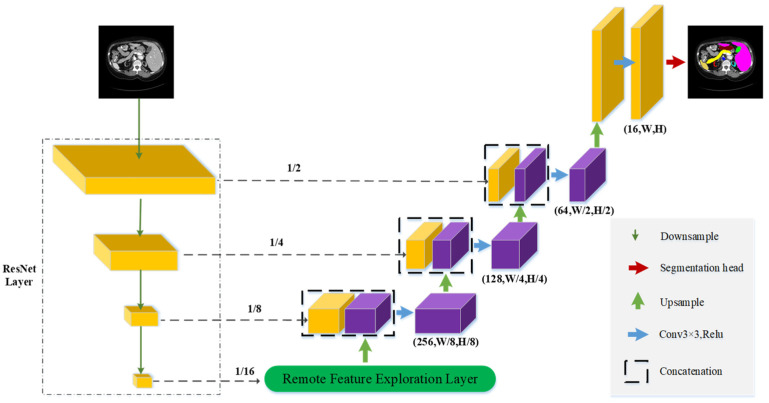
Overview of the RFE-UNet.

As shown in Figure 1, we describe the overall network structure of RFE-UNet, which consists of two processes: downsampling and upsampling. Among them, downsampling is the process of extracting foreground information and eliminating interference information. Upsampling is the process of restoring the downsampled feature map to a label map. The residual structure is employed during downsampling to extract important information from the feature map, as it contains valuable foreground information that can aid in generating deeper features. Specifically, this structure is used to learn the edge information of the focal region in the feature map. As the network becomes deeper, the proportion of foreground information in the feature map increases. To better integrate different types of foreground information, the remote feature exploration layer is proposed. This layer can leverage remote elements to help generate detailed information at the local level. For the upsampling layer, we continue to use the previous practice of upsampling deep features, and then fuse them with the features of the downsampling layer. This is to mitigate the negative impact of information loss on element discrimination during downsampling. We then describe the detailed process of each structure in detail.

### 3.1. ResNet Layer

**Figure 2:**
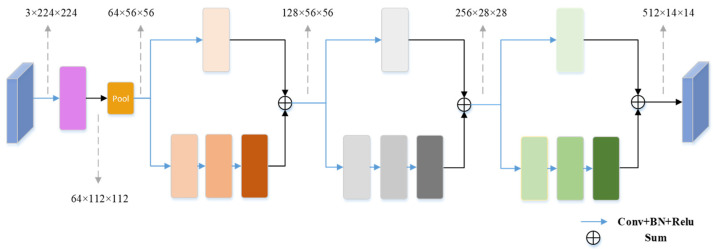
ResNet Layers.

For the ResNet layer, we mainly use the convolution operation and the jump connection operation. As shown in Figure 2, for the input original image, its image size is 3 × 224 × 224. Since the background information dominates the feature map, and the foreground information is only a small portion, the convolution operation is utilized to improve the learning of the foreground information within the image region block. In the process of downsampling, the network will inevitably lose some important foreground information, so we use the jump connection operation to repeatedly input the shallow information into the deep network to alleviate the information loss caused by this situation. The output feature map size is 512 × 14 × 14 after undergoing a sequence of convolutional operations. In order to make the network reach convergence faster, BatchNorm is added after the convolution operation to reduce the training time of the network. In addition, this paper also uses the ReLU activation function to improve the ability of the network to learn the nonlinear relationship between elements.

### 3.2. Remote Feature Exploration Layer

Before we introduce the working principle of the remote feature exploration layer in detail, let us explain some symbolic meanings. At this stage, the remote feature exploration layer is fed with the feature map generated from ResNet layers. The size of the feature map at this point measures 512 × 14 × 14. Since the Remote feature exploration layer mainly operates in the spatial dimension of the feature map, we assume that the size of the feature map F input to the remote feature exploration layer is 1 × 4 × 4. In this context, the feature map is described as having a spatial dimension of 4 × 4 and a single channel, denoted by the number 1.

**Figure 3:**
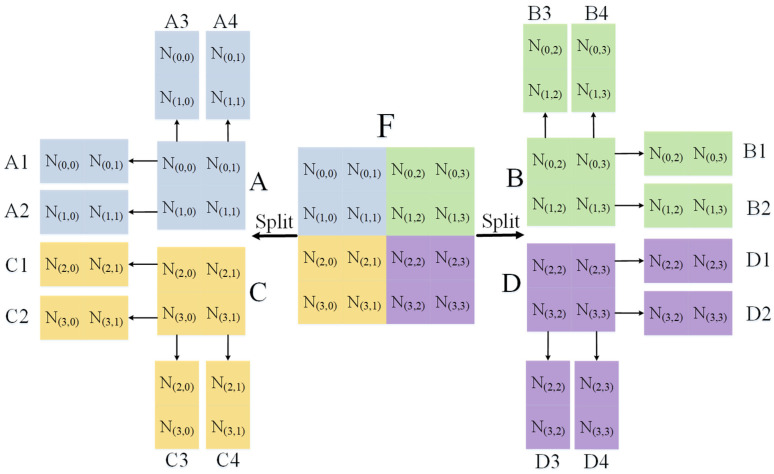
For the feature graph F, $N_{\left(0,0\right)}$......$N_{\left(3,3\right)}$, respectively, represent the specific element value at a certain point. Different letters represent different feature blocks.

As shown in Figure 3, the size of our input feature map $F=\left[\begin{array}{cc} \begin{array}{cc} N_{\left(0,0\right)} & N_{\left(0,1\right)}\\ N_{\left(1,0\right)} & N_{\left(1,1\right)} \end{array} & \begin{array}{cc} N_{\left(0,2\right)} & N_{\left(0,3\right)}\\ N_{\left(1,2\right)} & N_{\left(1,3\right)} \end{array}\\ \begin{array}{cc} N_{\left(2,0\right)} & N_{\left(2,1\right)}\\ N_{\left(3,0\right)} & N_{\left(3,1\right)} \end{array} & \begin{array}{cc} N_{\left(2,2\right)} & N_{\left(2,3\right)}\\ N_{\left(3,2\right)} & N_{\left(3,3\right)} \end{array} \end{array}\right]$ is 1 × 4 × 4. In order to achieve the goal of remote element-assisted local feature generation, we cut the feature map F in the spatial dimension to obtain feature maps A,B,C,D of the same size. The size of feature map $A=\left[\begin{array}{cc} N_{\left(0,0\right)} & N_{\left(0,1\right)}\\ N_{\left(1,0\right)} & N_{\left(1,1\right)} \end{array}\right]$, $B=\left[\begin{array}{cc} N_{\left(0,2\right)} & N_{\left(0,3\right)}\\ N_{\left(1,2\right)} & N_{\left(1,3\right)} \end{array}\right]$, $C=\left[\begin{array}{cc} N_{\left(2,0\right)} & N_{\left(2,1\right)}\\ N_{\left(3,0\right)} & N_{\left(3,1\right)} \end{array}\right]$, $C=\left[\begin{array}{cc} N_{\left(2,2\right)} & N_{\left(2,3\right)}\\ N_{\left(3,2\right)} & N_{\left(3,3\right)} \end{array}\right]$ are 1 × 2 × 2. For feature map A, we extract the elements of each row in the row dimension to obtain feature maps $A1=\left[\begin{array}{cc} N_{\left(0,0\right)} & N_{\left(0,1\right)} \end{array}\right]$ and $A2=\left[\begin{array}{cc} N_{\left(1,0\right)} & N_{\left(1,1\right)} \end{array}\right]$, respectively, and the dimensions of feature maps A1 and A2 are 1 × 2 × 2, respectively. Then we extract each column element from the column dimension to obtain feature maps $A3=\left[\begin{array}{c} N_{\left(0,0\right)}\\ N_{\left(1,0\right)} \end{array}\right]$ and $A4=\left[\begin{array}{c} N_{\left(0,1\right)}\\ N_{\left(1,1\right)} \end{array}\right]$, respectively. At this point, the size of feature maps A3 and A4 are 1 × 2 × 1. For feature map B, we also follow the same procedure to obtain feature maps B1, B2, B3, and B4, where the size of feature maps $B1=\left[\begin{array}{cc} N_{\left(0,2\right)} & N_{\left(0,3\right)} \end{array}\right]$ and $B2=\left[\begin{array}{cc} N_{\left(1,2\right)} & N_{\left(1,3\right)} \end{array}\right]$ are 1 × 1 × 2, and the size of feature maps $B3=\left[\begin{array}{c} N_{\left(0,2\right)}\\ N_{\left(1,2\right)} \end{array}\right]$ and $B4=\left[\begin{array}{c} N_{\left(0,3\right)}\\ N_{\left(1,3\right)} \end{array}\right]$ are 1 × 2 × 1. Similarly, after the same operation, we can obtain feature maps $\left\{C1=\left[\begin{array}{cc} N_{\left(2,0\right)} & N_{\left(2,1\right)} \end{array}\right],\;C2=\left[\begin{array}{cc} N_{\left(3,0\right)} & N_{\left(3,1\right)} \end{array}\right],C3=\left[\begin{array}{c} N_{\left(2,0\right)}\\ N_{\left(3,0\right)} \end{array}\right],\;C4=\left[\begin{array}{c} N_{\left(2,1\right)}\\ N_{\left(3,1\right)} \end{array}\right]\right\}$ and $\left\{D1=\left[\begin{array}{cc} N_{\left(2,2\right)} & N_{\left(2,3\right)} \end{array}\right],D2=\left[\begin{array}{cc} N_{\left(3,2\right)} & N_{\left(3,3\right)} \end{array}\right],D3=\left[\begin{array}{c} N_{\left(2,2\right)}\\ N_{\left(3,2\right)} \end{array}\right],D4=\left[\begin{array}{c} N_{\left(2,3\right)}\\ N_{\left(3,3\right)} \end{array}\right]\right\}$ from feature map C and feature map D, respectively.

**Figure 4:**
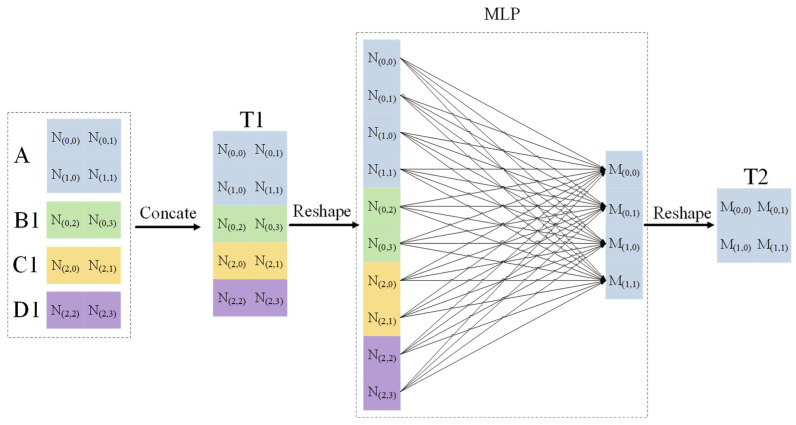
With feature graph A as the base unit, remote elements B1, C1, and D1 are used to assist A to generate a new feature graph.

Next, we take the feature map A as the base unit and describe in detail how the remote elements assist in the generation of local features. As shown in Figure 4, we fuse feature map $A\in R^{1\times 2\times 2}$ and strip feature maps $B1\in R^{1\times 1\times 2}$, $B2\in R^{1\times 1\times 2}$, and $B3\in R^{1\times 1\times 2}$ in the channel dimension to form feature map $T1\in R^{1\times 5\times 2}$. Then, we tile the feature map T1, stretch it into two-dimensional data, and input it into the fully connected layer for element correlation calculation, as shown in the following:

(1) $$M_{\left(0,0\right)}=w_{\left(0,0\right)}N_{\left(0,0\right)}+w_{\left(1,0\right)}N_{\left(0,1\right)}+\cdots +w_{\left(8,0\right)}N_{\left(2,2\right)}+w_{\left(9,0\right)}N_{\left(2,3\right)},$$

(2) $$M_{\left(0,1\right)}=w_{\left(0,1\right)}N_{\left(0,0\right)}+w_{\left(1,1\right)}N_{\left(0,1\right)}+\cdots +w_{\left(8,1\right)}N_{\left(2,2\right)}+w_{\left(9,1\right)}N_{\left(2,3\right)},$$

(3) $$M_{\left(0,2\right)}=w_{\left(0,2\right)}N_{\left(0,0\right)}+w_{\left(1,2\right)}N_{\left(0,1\right)}+\cdots +w_{\left(8,2\right)}N_{\left(2,2\right)}+w_{\left(9,2\right)}N_{\left(2,3\right)},$$

(4) $$M_{\left(0,3\right)}=w_{\left(0,3\right)}N_{\left(0,0\right)}+w_{\left(1,3\right)}N_{\left(0,1\right)}+\cdots +w_{\left(8,3\right)}N_{\left(2,2\right)}+w_{\left(9,3\right)}N_{\left(2,3\right)},$$

where $M_{\left(0,0\right)}$, $M_{\left(0,1\right)}$, $M_{\left(0,2\right)}$, and $M_{\left(0,3\right)}$ represent the values of the elements at a specific location generated, and $w_{\left(i,j\right)\left(0\leq i\leq 9,0\leq j\leq 3\right)}$ represents the specific parameter values. The formula shows that the generation of $M_{\left(0,0\right)}$, $M_{\left(0,1\right)}$, $M_{\left(0,2\right)}$, and $M_{\left(0,3\right)}$ not only uses the local features of A but also incorporates the elements from the remote feature maps B1, C1, and D1. In this way, we can achieve the generation of remote element-assisted local detail features.

**Figure 5:**
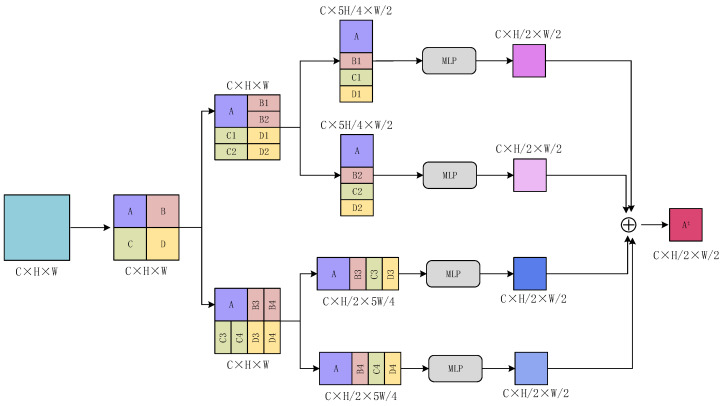
Using A as an example, remote elements assist in the detailed flow diagram of local feature generation.

From Figure 4, we know how the remote elements assist in the generation of local features. Then, as depicted in Figure 5, feature map A can be integrated with other spatial elements in four distinct manners to produce a novel feature map. Following this, an MLP operation and feature fusion are employed on the generated feature maps to obtain the ultimate feature map $A^{t}$. This elucidates the usage of feature map A as the fundamental building block. In the same way, we can generate new feature maps $B^{t}$, $C^{t}$, and $D^{t}$ with feature maps B, C, and D as the base unit, respectively. Finally, we can recreate the new feature maps $A^{t}$, $B^{t}$, $C^{t}$, $D^{t}$ in the length and width dimensions to generate feature maps with the same size as the input.

### 3.3. Decoder of RFE-UNet

Similar to the U-Net [1] architecture, the RFE-UNet proposed in this study also employs a U-shaped structure for obtaining prediction maps. As illustrated in Figure 1, we utilize an extended convolution kernel to enlarge the size of the feature map after feeding the downsampled output feature map into the decoder. Due to the feature extraction process during downsampling, some crucial information may be lost in the network. To address this, we utilize skip connections to fuse the feature maps in both the encoder and decoder to minimize any negative impact on the prediction map generation. The number of skip connections was reduced in recent network architectures such as TransUNet [26] and SwinUNet [29]. However, experimental findings indicate that the recovery of the prediction map improves as the number of skipped connections increases. Therefore, we directly use the same number of skipped connections as TransUNet [26] in this paper.

## 4. Experiments and Analysis

Section 4.1 is devoted to present the datasets used in the experiment. This includes a detailed breakdown of the data quantity, access channels, and specific prospect information for the two kinds of datasets. In order to confirm the credibility and reliability of the experiment, a comprehensive account of the experimental details are provided in Section 4.2, which details the experimental environment and various superparameters employed in this study. Furthermore, in Section 4.3 and Section 4.4, we provide an in-depth explanation of the loss functions and evaluation metrics used in the experiment, as well as the rationale behind their use. Section 4.5 presents the comparative and ablation test results, and Section 4.6 details the findings of the analytical study. The visualization results are discussed in Section 4.7. To summarize, Section 4 utilizes specific experimental results to validate the innovation points presented in this paper. It is important to note that no patients were harmed during the experiments, and patient identities in the experimental image data were removed.

### 4.1. Datasets

In order to estimate the effects of our proposed method, a significant number of experiments were implemented on a public organ segmentation dataset (Synapse) and another segmentation dataset (ours). The public dataset used was the Synapse multi-organ segmentation dataset, provided by the authors of TransUNet [26]. It is available for access at https://www.synapse.org/#!Synapse:syn3193805/wiki/217789 (accessed on 1 November 2022). The multi-organization dataset (MOD) was derived from CT scans of different patients. The MOD dataset was created to explore the performance of methods for multi-organ segmentation. The MOD dataset comes from a previous collection of the hospital, in which all image data were amended to protect patient identity, collection time, and other privacy information. This paper respects the ethics of medical data; the research methods and experiments adopted do not cause any harm to patients and do not involve any conflicts of interest or other issues. We will consider publishing the MOD dataset for further study by scholars at a later date. Below, there will be a detailed explanation regarding the quantity of datasets utilized for both training and testing in the laboratory.

The Synapse dataset is a collection of 3779 clinical CT images of the abdominal region, taken from 30 cases using CT scanners. Each image depicts one or more of the eight abdominal organs; namely, the aorta, gallbladder, left kidney, right kidney, liver, pancreas, spleen, and stomach. The dataset was split into two sets; one for training consisting of 18 samples, and another for testing consisting of 12 samples, in line with previous studies [26,29].

The multi-organization dataset (MOD) is a dataset obtained from Xinjiang Urumqi Hospital of Traditional Chinese Medicine (Hongshan), China. The dataset includes 430 clinical images from 145 patients acquired from CT scanners. The CT images are a series of routine scanning slices from the upper abdomen to the mid abdomen, with a slice thickness of 7 mm. The 430 images were labeled by LabelImg, and the labeled samples were approved by professional doctors. Each sample’s foreground information comprises eight organs: the aorta, gallbladder, left kidney, right kidney, liver, pancreas, spleen, and stomach. The 430 images were split randomly into a training set and a testing set, with a ratio of 7:3.

### 4.2. Implementation Details

For this experiment, we kept the original feature map resolution fixed at 224 × 224 and used a batch size of 24, except for cases where special instructions required otherwise. We trained our model using the SGD optimizer, setting the learning rate to 0.01, the momentum to 0.9, and the weight decay rate to 0.0001. The default training iteration number was 20,000. All experiments were conducted on Python 3.6 and Torch 1.6.0, with two NVIDIA Tesla V100 GPUs used for parallel training and testing.

### 4.3. Loss Function

Since one loss function often does not accurately reflect good or bad performance of medical image segmentation, a mixture of two loss functions (cross-entropy loss and dice loss) were used in our network model, which is in line with many current segmentation methods.

Cross-entropy loss is a popular loss function for medical image segmentation, especially multi-classification problems. The smaller the value of cross-entropy is, the better the model prediction will be, with Equation (5) as follows:

(5) $${ℒ}_{CrossEntoryLoss}=-\sum_{x}\left(p\left(x\right)logq\left(x\right)\right),$$

where p(x) stands for ground-truth label and q(x) stands for predictive value.

Dice loss is the evolution of the dice coefficient. The dice coefficient is a metric function that evaluates the similarity of two samples, where the larger value means the predictive label is more similar to the ground-truth label. Equation (6) for the dice co-efficient is as follows:

(6) $$Dice=\frac{2\left|X\cap^{\;}Y\right|}{\left|X\right|+\left|Y\right|},$$

where $\left|X\cap^{\;}Y\right|$ denotes the number of elements in the intersection between X and Y, and |X| and |Y| denote the number of elements in X and Y, respectively. The dice loss is calculated as shown in Equation (7):

(7) $${ℒ}_{Dice}=1-Dice=1-\frac{2\left|X\cap^{\;}Y\right|}{\left|X\right|+\left|Y\right|}$$

where X stands for ground-truth label and Y stands for predictive value.

The mixture-loss of the network is as follows in Equation (8):

(8) $${ℒ}_{total-loss}=\lambda_{1}*{ℒ}_{Dice}+\lambda_{2}*{ℒ}_{CrossEntoryLoss}$$

where $\lambda_{1}$ and $\lambda_{2}$ are the weighting coefficients of the cross-entropy loss and dice loss, respectively. As a matter of experience, we set $\lambda_{1}\;$= $\lambda_{2\;}$= 0.5 in this paper.

### 4.4. Evaluation Metrics

The dice similarity coefficient (*DSC*) is a kind of set similarity metric, usually used to evaluate the degree of similarity with a value range of 0–1. *DSC* (%) is calculated as shown in Equation (9):

(9) $$DSC=\frac{2\left|X\cap^{\;}Y\right|}{\left|X\right|+\left|Y\right|}\times 100\%$$

where X and Y represent the group truth and prediction, respectively.

The Hausdorff distance evaluates the similarity of any two sets in metric space. *HD*95 (mm) can be described as a discrete numerical value obtained by quantizing 95% of the maximum difference between the predicted value and the actual value. The methods used to calculate *HD*95 (mm) are presented in Equation (10):

(10) $$HD95=max_{k95\%}\left[d\left(X,Y\right),d\left(Y,X\right)\right]$$

where X and Y represent the group truth and prediction, respectively.

### 4.5. Experimental Results

Table 1 and Table 2 display the findings of the experiments conducted on the Synapse and MOD datasets. The superior performance value is indicated by the bold text in the tables and will not be explicitly discussed further in the following text.

As shown in Table 1, the performance of the traditional CNN is still better, and the performance of Att-UNet exceeds that of TransUNet. Nonetheless, our methodology exhibits remarkable superiority compared to CNN-based techniques such as U-Net, attention-mechanism-based methods such as Att-UNet, and transformer-based approaches such as TransUNet, among others. On the Synapse dataset, the average DSC (%) of our method (RFE-UNet) reached 79.77%, obtaining the optimal average DSC (%). Compared with other CNN-based methods, RFE-UNet obtained optimal results for four organs (left kidney, right kidney, pancreas and stomach). Our method can not only calculate the correlation of elements at short distances, but it can also model elements at long distances. Therefore, RFE-UNet is better than the CNN model, which only extracts local feature information. Our method outperforms other transformer methods in three organs (left kidney, right kidney, and liver). The reason is that the relevance of some local elements is inevitably ignored in the feature extraction stage of a model based on a transformer, resulting in a certain degree of information loss. In general, the performance of RFE-UNet is more accurate, and it is better able to consider the correlation of different categories of prospect information.

Table 2 demonstrates that on the MOD dataset, our approach (RFE-UNet) achieved mean DSC (%) and *HD*95 (mm) values of 75.12% and 7.43, respectively. This represents a 0.54% improvement in DSC (%) performance compared to the baseline (U-Net). Notably, our methodology performs exceptionally well in the segmentation of the left kidney. In this new dataset, it is clear that the CNN-based network segmentation performance is better than the transformer-based network. Compared with the method based on the CNN, we obtained the optimal value of the left kidney. In comparison to the transformer-based method, we obtained the optimal values for four organs (aorta, gallbladder, left kidney, and stomach). Compared with other methods, the overall segmentation effect of our method is optimal. This is because the accepting domain of the convolutional block in the CNN-based method is limited and the long-term dependency cannot be calculated. The transformer-based approach tends to model the global context rather than the local details. In contrast, RFE-UNet can complete local and global modeling at the same time, which strengthens the richness and relevance of the extracted sample features, and leads to excellent performance of image segmentation.

### 4.6. Analytical Study

**Experimental analysis of ablation based on the RFE-Layer**.

As shown in Figure 5, we try to realize the information exchange of remote features only by using a single block feature diagram (for example, one of A, B, C, or D, corresponding to “REF-A”, “REF-B”, “REF-C”, “REF-D”) as the basic unit, with the experimental results shown in Table 3 and Table 4.

As can be seen from Table 3, the effect of “REF-A”, “REF-C”, and “REF-D” on the Synapse dataset is better than that of four blocks working together (RFE-Layer). This is because not all labeled images in this dataset contain all the organs, and some labels contain only a few organs and occupy a small area. Therefore, when only the single block feature map is used as the basic unit, the segmentation of organs with a small area is advantageous. On the MOD dataset (as shown in Table 4), since all the labeled images of this dataset contain most organ categories, the segmentation effect is better when four blocks work together (RFE-Layer).

**Experimental analysis of ablation based on different input resolution.**

By default, the input resolution for RFE-UNet is set at 224 × 224. However, we conducted experiments with RFE-UNet trained at resolutions of 256 × 256 on both the Synapse and MOD datasets, as illustrated in Table 5 and Table 6. Despite the increase in input resolution, the patch size remains constant at 16, leading to an increase in the sequence length of the input remote feature exploration layer. The increase in DSC (%) on Synapse and MOD datasets is 0.7% and 1%, respectively, compared to the 224 × 224 input size, and at the same time leads to a computational cost increase. The data shown in Table 5 and Table 6 illustrate that a segmentation performance increase can be achieved by improving image resolution. However, higher resolution also means that we will pay more computing resource for an increase in average DSC (%). Given our limited GPU memory resources, we opted to conduct experiments at the resolution of 224 × 224 to establish the validity and reliability of RFE-UNet while taking into account the computational costs involved. In Table 5 and Table 6, we show the mean DSC (%) of different input image resolutions on the two datasets and the segmentation accuracy of eight organs, respectively. The influence of resolution on the performance of image segmentation is consistent with what is reported by the proposed study.

### 4.7. Visualizations

**Figure 6:**
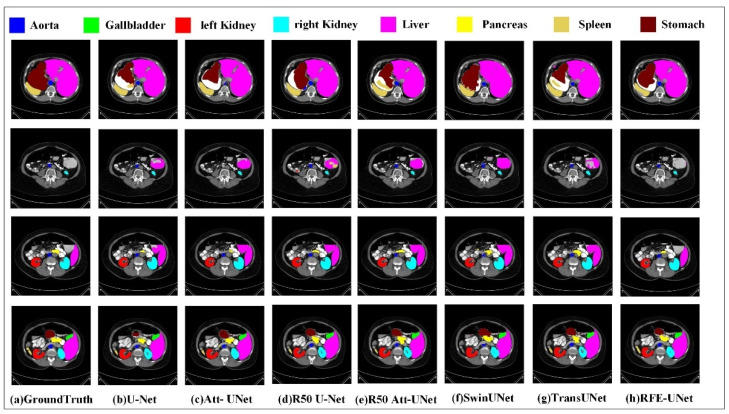
Results of qualitative experiments on the Synapse dataset.

**Figure 7:**
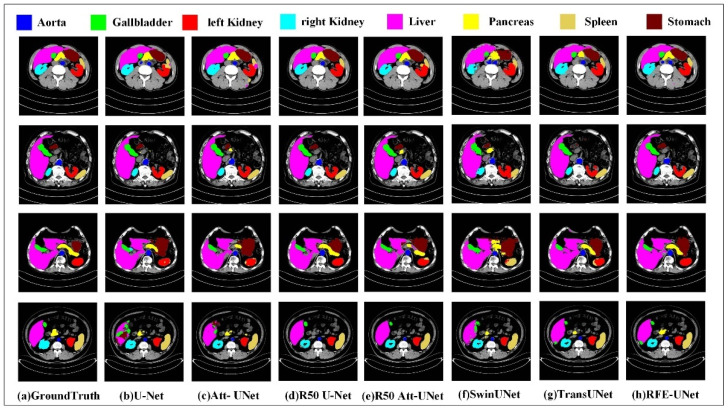
Results of qualitative experiments on the MOD dataset.

Figure 6 and Figure 7 display a qualitative comparison of the segmentation performance of RFE-UNet on the Synapse and MOD datasets.

The visualized results on the Synapse dataset are shown in Figure 6. We can see from the analysis of Figure 6 that: (1) CNN-based methods (such as U-Net) show weaker performance when segmenting organs that are further apart than transformer-based methods (such as TransUNet). For example, U-Net and R50 Att-UNet show significant false positives for the liver in the second and third lines, while the segmentation of the stomach in the first line and the spleen in the fourth line is incomplete. The main reason is that when the CNN uses convolution kernels for feature extraction, it mainly focuses on the correlation modeling of local features while ignoring the remote correlation of samples. Therefore, the CNN class model makes it easy to lose global information, resulting in unsatisfactory segmentation results. (2) The transformer-based approach improves the situation to some extent. For example, TransUNet shows less over-segmentation and under-segmentation than the pure CNN method because of its global element correlation modeling ability. However, the transformer inevitably ignores the importance of local details when modeling global elements. Since the structure of medical images is complex, local details and global correlation are very important for segmentation results. (3) In comparison with other network models, our RFE-UNet segmentation has better results. The main reason is that RFE-UNet uses the proposed remote feature exploration layer to complete the interaction between the elements of the near and far, and to realize the joint modeling of local and global correlations. The qualitative experimental results in Figure 6 show that our RFE-UNet has excellent segmentation performance considering both local and global correlations.

On the MOD dataset, Figure 7 shows that: (1) Based on pure CNN methods, such as U-Net and Att-UNet, it is more likely to lead to over-segmentation of organs (e.g., the spleen in the first line, the stomach and gallbladder in the fourth line, etc.) or under-segmentation (e.g., the liver in the fourth line). The reason is that although the CNN-based method has strong local modeling ability, the receptive field of the convolution calculation is local. Therefore, these methods cannot effectively capture the global element correlation in the sample and lose the long-distance dependence relationship. (2) The addition of the transformer to TransUNet improves the situation to some extent. In terms of long-distance correlation modeling, TransUNet has a stronger coding capability than the CNN. However, the overall segmentation effect of the SwinUNet method based on a pure transformer architecture is not ideal. This is because the transformer focuses on modeling global element associations and ignores local element associations. In medical segmentation tasks, organs often appear in the form of regional blocks, the connection between target organs is very close, and there are size differences between organs. Therefore, the lack of local modeling and global modeling may lead to unsatisfactory segmentation results. (3) Compared with other methods, the remote feature exploration layer proposed in RFE-UNet can use remote elements to assist the generation of local features and realize the joint modeling of local and global correlations, so it has a better segmentation effect. Notably, the segmentation of the stomach in the third row exhibits no false positives, and the segmentation of the left kidney is notably superior to the other methods. The comprehensive results in Figure 7 show that the segmentation results of RFE-UNet are smoother, the target structure is more complete, and it is closer to the tag. This is due to the fact that we consider the importance of both local and global dependencies to perform segmentation tasks.

## 5. Conclusions

In this paper, we proposed the remote feature exploration layer based on the ad-vantages and disadvantages of the CNN and the transformer. The CNN uses convolution kernels for feature extraction, which affords the network a strong local modeling ability; however, this feature extraction method also greatly reduces the remote modeling ability of the network. Unlike the CNN, the transformer can model global elements, but it inevitably loses some of the local details. Therefore, we propose a remote feature exploration module. This module assists the network in generating local details by using remote elements. Through this feature-learning method, the network develops the joint modeling ability of local and remote elements to a certain extent. Experiments on Synapse and MOD datasets showed that our model has better segmentation performance compared with convolutional series segmentation models, convolutional transformer hybrid segmentation models, and pure transformer segmentation models. In the future, we will explore how to convert static blocks in the remote feature exploration layer into adaptive, dynamically selected blocks.

## Figures and Tables

**Table 1:** The results from the Synapse dataset, along with the DSC (%) for each individual class.

Method	DSC (%)	*HD*95 (mm)	Aorta	Gallbladder	Kidney (L)	Kidney (R)	Liver	Pancreas	Spleen	Stomach
V-Net [34]	68.81	-	75.34	51.87	77.10	80.75	87.84	40.05	80.56	56.98
DARR [32]	69.77	-	74.74	53.77	72.31	73.24	94.08	54.18	89.90	45.96
R50 U-Net [31]	74.68	36.87	84.18	62.84	79.19	71.29	93.35	48.23	84.41	73.92
R50 Att-UNet [31]	75.57	36.97	55.92	63.91	79.20	72.71	93.56	49.37	87.19	74.95
U-Net [1]	76.85	39.70	89.07	**69.72**	77.77	68.60	93.43	53.98	86.67	75.58
UNet++ [12]	78.13	25.65	89.27	62.35	83.00	78.98	94.53	56.70	85.99	74.20
UNet3+ [33]	73.81	30.82	86.32	59.06	79.16	71.26	93.13	46.56	84.94	70.08
Att-UNet [41]	77.77	36.02	**89.55**	68.88	77.98	71.11	93.57	58.04	87.30	75.75
R50 ViT [22]	71.29	32.87	73.73	55.13	75.80	72.20	91.51	45.99	81.99	73.95
ViT [22]	61.50	39.61	44.38	39.59	67.46	62.94	89.21	43.14	75.45	69.78
TransUNet [26]	77.48	31.69	87.23	63.13	81.87	77.02	94.08	55.86	85.08	75.62
SwinUNet [29]	79.13	**21.55**	85.47	66.53	83.28	79.61	94.29	56.58	**90.66**	76.60
MT-UNet [42]	78.59	26.59	87.92	64.99	81.47	77.29	93.06	**59.46**	87.75	76.81
UCTransNet [28]	79.11	25.08	88.58	64.34	82.93	75.93	**95.42**	56.77	88.20	**80.67**
SepViT [43]	77.77	30.37	88.36	67.49	80.97	77.36	93.21	53.27	88.31	73.21
RFE-UNet (Ours)	**79.77**	21.75	87.32	65.40	**84.18**	**81.92**	94.34	59.02	89.56	76.45

**Table 2:** The results on the MOD dataset, along with the DSC (%) for each individual class.

Method	DSC (%)	*HD*95 (mm)	Aorta	Gallbladder	Kidney (L)	Kidney (R)	Liver	Pancreas	Spleen	Stomach
R50 U-Net [31]	73.64	5.10	88.82	87.12	62.07	48.54	93.81	59.12	74.81	74.84
R50 Att-UNet [31]	74.62	**4.46**	**88.87**	**87.29**	62.01	50.78	**95.19**	56.51	78.32	**78.00**
U-Net [1]	74.58	6.34	88.07	85.81	62.38	52.99	93.94	63.65	75.85	73.91
Att-UNet [42]	74.56	5.55	88.40	85.93	64.12	**53.04**	94.36	63.25	73.89	73.49
TransUNet [26]	73.35	7.32	87.90	83.83	62.72	50.87	94.38	54.86	**79.14**	73.13
SwinUNet [29]	70.90	10.31	74.43	74.76	66.20	52.08	90.33	**69.89**	71.09	68.43
UCTransNet [28]	73.10	6.48	87.50	82.11	63.82	51.72	93.75	58.25	73.82	73.83
RFE-UNet (Ours)	**75.12**	7.43	88.11	84.72	**66.43**	50.91	94.27	63.98	77.34	75.21

**Table 3:** Results of experiments based on the RFE-Layer on the Synapse dataset.

Model	DSC (%)	*HD*95 (mm)	Aorta	Gallbladder	Kidney (L)	Kidney (R)	Liver	Pancreas	Spleen	Stomach
RFE-A	**80.64**	22.44	87.41	64.75	84.84	82.78	94.50	**62.77**	88.70	79.35
RFE-B	79.58	20.81	87.71	58.43	**85.95**	81.61	94.48	59.28	**90.33**	78.88
RFE-C	79.90	25.99	87.54	66.07	82.52	79.90	94.27	59.71	89.23	**79.95**
RFE-D	80.16	**20.67**	**87.76**	**66.96**	83.63	81.16	**95.08**	59.81	89.05	77.86
RFE-Layer	79.77	21.75	87.32	65.40	84.18	**81.92**	94.34	59.02	89.56	76.45

**Table 4:** Results of experiments based on the RFE-Layer on the MOD dataset.

Model	DSC (%)	*HD*95 (mm)	Aorta	Gallbladder	Kidney (L)	Kidney (R)	Liver	Pancreas	Spleen	Stomach
RFE-A	72.60	6.44	89.65	83.95	63.66	49.22	94.33	57.70	70.94	71.39
RFE-B	73.14	**5.82**	89.60	84.98	63.24	49.53	94.53	57.87	73.22	72.12
RFE-C	73.13	5.94	89.60	85.62	63.53	50.27	**94.68**	56.60	72.60	72.13
RFE-D	73.19	6.04	**89.71**	**86.06**	62.56	49.51	94.64	57.29	73.01	72.69
RFE-Layer	**75.12**	7.43	88.11	84.72	**66.43**	**50.91**	94.27	**63.98**	**77.34**	**75.21**

**Table 5:** Results of experiments with different input resolutions on the Synapse dataset.

Resolution	DSC (%)	*HD*95 (mm)	Aorta	Gallbladder	Kidney (L)	Kidney (R)	Liver	Pancreas	Spleen	Stomach
224	79.77	**21.75**	**87.32**	65.40	**84.18**	**81.92**	94.34	59.02	**89.56**	76.45
256	**80.47**	23.19	86.77	**68.85**	82.77	81.21	**94.63**	**60.65**	89.33	**79.53**

**Table 6:** Results of experiments with different input resolutions on the MOD dataset.

Resolution	DSC (%)	*HD*95 (mm)	Aorta	Gallbladder	Kidney (L)	Kidney (R)	Liver	Pancreas	Spleen	Stomach
224	75.12	7.43	88.11	84.72	66.43	**50.91**	94.27	63.98	**77.34**	75.21
256	**76.12**	**6.57**	**88.45**	**85.26**	**69.88**	50.56	**94.32**	**68.95**	75.15	**76.38**

## Data Availability

The Synapse dataset is openly available at: https://www.synapse.org/#!Synapse:syn3193805/wiki/217789 (accessed on 9 December 2022). The MOD dataset will be made public in the future and available links will be published in the future.
